# Supplementary material for: Supplemental Ferulic Acid Inhibits Total Body Irradiation-Mediated Bone Marrow Damage, Bone Mass Loss, Stem Cell Senescence, and Hematopoietic Defect in Mice by Enhancing Antioxidant Defense Systems
Source: Antioxidants (Basel). 2021 Jul 28;10(8):1209. doi: 10.3390/antiox10081209 (PMC8388974; doi:10.3390/antiox10081209)
Supplement: Supplementary file 1 [file antioxidants-10-01209-s001.zip › antioxidants-1293011-supplementary.pdf]

Supporting Information

Table S1. Sequences of primers used to analyze the expression of adipogenesis-related genes

**Genes**	**Forward primers**	**Reverse primers**	**NCBI reference sequence number**
C/EBPα	tcctaccgagtagggggagc	gcccgagaggaagcaggaat	NM_001287514.1
PPARγ	acggttgatttctccagcat	ggacgcaggctctactttga	XM_036165927.1
apM1	gtcagtggatctgacgacaccaa	atgcctgccatccaacctg	NM_009605.5
GAPDH	gacggccgcatcttcttg	cacaccgaccttcaccat	XM_017321385.1

Supplemental figures and legends

Figure S1.

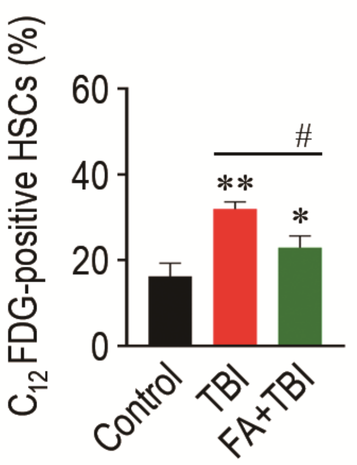

Figure S1. Flow cytometric analysis showing the mean percentage of C_{12}FDG-positive HSCs in BM of mice groups at 30 days post-TBI (*n* = 4).

Figure S2.

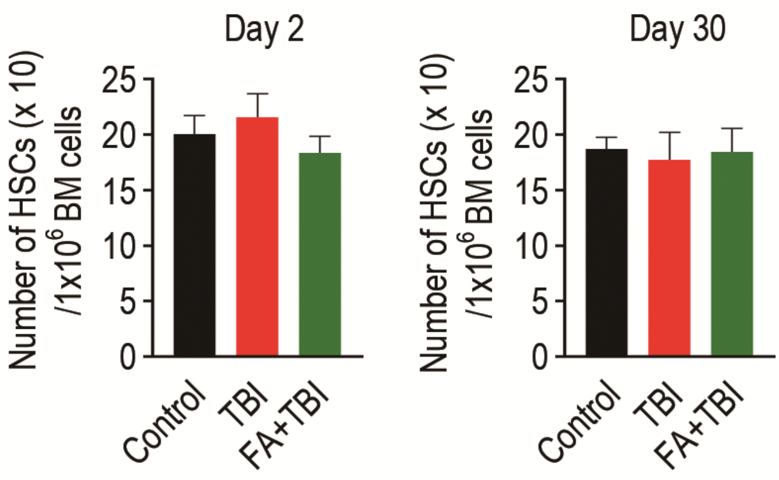

Figure S2. Flow cytometric analysis showing the mean number of HSCs in BM of mice groups at 2 and 30 days post-TBI (*n* = 4).
